# A systematic review on soil microbial shifts under drought stress: a climate-smart agriculture perspective

**DOI:** 10.1093/femsec/fiag044

**Published:** 2026-04-22

**Authors:** Ndivhuwo Ramatsitsi, Alen Manyevere

**Affiliations:** Department of Agronomy, University of Fort Hare, Private Bag X1314, Alice, 5700, South Africa; Department of Agronomy, University of Fort Hare, Private Bag X1314, Alice, 5700, South Africa

**Keywords:** climate change, drought-resistant, microbial diversity, resilience, water scarcity

## Abstract

Major shifts in temperature and rainfall patterns resulting from climate change are projected to continue increasing intensely over the course of the century. Ecosystems’ functionality and well-being of above-ground plant community are all significantly impacted by soil microbes’ response to these shifting abiotic stresses. With an emphasis on improving their usefulness in climate-smart agriculture (CSA), we reviewed how soil bacteria and fungi tolerate drought stress and improve plant development under water shortage conditions. This systematic analysis used Preferred Reporting Items for Systematic Reviews and Meta-Analyses (PRISMA) to elaborate how microbe-based solutions could be incorporated in CSA. A total of 31 articles satisfied the inclusion criteria. The review demonstrated that soil microbial diversity and abundance are considerably altered by drought stress, improve resilience of plants, and soil functionality. There was a further observation of high microbial community in the endosphere, and rhizosphere as compared to bulk soil; a clear indication of plants’ potential to facilitate soil microbial assemblages. Evidently, plants under drought conditions exude metabolites that stimulate drought-tolerant microbes; that in-turn promote the plants’ tolerance to drought. Accordingly, this remarkable synergy between microbes and plants could help forecast how agroecosystems would function in the face of climate change.

## Teaser

The paper evaluated how drought resistant bacteria and fungi promote plant growth and development under water shortage, in accordance with several sustainable development goals and emphasis on climate-smart agriculture.

## Introduction

Global warming is causing drastic changes in temperature and rainfall regimes, and these changes are predicted to get exacerbated over the next century (Tang et al. 2025). Increased drought-related stress from global warming and climate change has detrimental impacts on plant functionality and survival because it induces metabolic dysfunction and inhibits photosynthetic carbon dioxide fixation, which lead to substantial growth declines and often premature plant death (Chaudhry and Sidhu 2022). Prospective projections indicate that due to water scarcity, this abiotic stress will reduce the yield of both agricultural (Junaid and Gokce 2024) and bioenergy crops in low-latitude regions (Heikonen et al. 2025). Although it is inconceivable to pinpoint the precise proportion of worldwide farms that utilize covered structures including plastic mulch or greenhouses, it is understood to constitute a negligible portion of all agricultural areas. It has been estimated that between 4–5 million hectares of land are under protected structures, representing a rather minute percentage of all arable land (Tong et al. 2024). Approximately 1.6 billion hectares were utilized for agricultural production in 2024 (Ritchie and Roser 2024). Of this, ∼40% is made up of drylands, which are home to more than two billion people. Most arid and semi-arid regions are anticipated to undergo modified rainfall patterns, encompassing variations in overall precipitation totals and fewer but more intense rainfall occurrences punctuated by extended dry spells (UN 2024). As such, there is a global dire need for climate-smart agriculture (CSA) strategies for improved productivity in water-scarce regions.

To raise awareness and restructure policy in response to climate change, CSA explores trade-offs and synergies between food security, adaptation, and mitigation. The goal of CSA is to enhance food security in the face of the current climatic challenges by readjusting and reshaping agricultural systems (Azadi et al. 2021). According to Engel and Muller (2016), CSA can increase agricultural output and revenue while fostering climate change resilience and reducing greenhouse gas emissions by implementing adaptation techniques. The main objective of CSA is to create resilient systems and high yields by utilizing modern agricultural technology and advancements. These include biologically derived inputs such as microbial inoculants and plant-based bio-stimulants, as well as sustainable land management practices such as conservation tillage, cover cropping, and the application of organic amendments. This is essential to meeting the food needs of a growing population and promoting the economic expansion needed to reduce poverty (Hussain et al. 2022). The close interaction between plants and micro-organisms can be exploited to achieve sustainable development goals (SDG) such as SDG No. 13 of “Climate action” and reduce negative environmental effects. Through root exudates, plants provide micro-organisms in the rhizosphere with carbon sources including sugars and amino acids, which promote microbial development (Hartmann et al. 2009, Vives-Peris et al. 2020). In exchange, by facilitating nutrient uptake, strengthening root development, providing ecological resilience, micro-organisms enhance plant health. Because micro-organisms foster growth, development and provide resilience to a variety of abiotic stressors, they can be employed to aid in climate change adaption (Ajala et al. 2022).

Abiotic stressors such as drought can simultaneously affect the physiology, growth, and productivity of plants while altering host-associated microbiomes. Prolonged water stress may remodel microbial networks, and favor recognized drought-tolerant micro-organisms in rhizospheres or roots, such as Actinobacteria, Chloroflexi, and Firmicutes (Bogati and Walczak 2022, Mazumder et al. 2025). Soil microbial communities are pivotal for ecosystem resilience, especially in the face of climate change. Plant–microbiome interactions play a significant role in enhancing ecosystem resilience by supporting plant health, restoring soil functionality, and acting as natural mediators of plant stress responses, enabling plants to recover from environmental stressors (Philippot et al. 2021). The purpose of this study was to systematically review how plants physiologically respond to water stress and concurrently modify belowground microbiomes. This review aims not only to summarize existing studies but to synthesize current knowledge by identifying key mechanisms underlying plant–microbe interactions under drought stress and evaluating their consistency across diverse agroecosystems. The following questions were the focus of this review: (i) What are plant-microbe abundance and diversity under drought stress? (ii) How do plant species facilitate soil microbial assemblages under water stress? and (iii) How do drought-resistant microbes influence plant adaptability to drought stress? This review adopts a broad, global perspective by integrating studies across diverse agroecosystems, plant species, and experimental conditions to identify general patterns in bacteria and fungi responses to drought stress within the context of CSA.

## Methodology

### Search strategy

Relevant articles were searched for, chosen, and collected by applying a methodical process that adhered to the Preferred Reporting Items for Systematic Reviews and Meta-Analyses (PRISMA) flow-diagram (Page et al. 2021). Research papers, reviews, and book chapters on plant-microbe interactions under drought stress were compiled from the following search databases: Science Direct, Google Scholar, and SCOPUS . “Drought-resistant microbes” and “climate-smart agriculture”, “distribution of drought-resistant microbes”, “effects of drought-resistant microbes on plant growth and productivity,” and “incorporation of drought-resilient microbes in climate-smart agriculture” were search terms used to collect research material on this topic from peer-reviewed scientific publications. A search protocol was created once the search queries were crafted to get pertinent data where the search terms were used to find the original material (Table 1). A formal meta-analysis was not conducted due to heterogeneity in experimental designs, microbial metrics, and reporting approaches across studies, which limited direct quantitative comparisons.

**Table 1:** Research protocol.

Items	Description
Research question	What are the plant-microbe interactions under drought stress?
Search database	Science Direct, Google Scholar, and SCOPUS
Search query 1	TITLE-ABS-KEY [(“drought-resistant” OR “drought-resilient” OR “drought-tolerant”) AND (“microbial communities” OR “microbial diversity” OR “microbial abundance” OR “microbial shift” OR “microbial functional groups”) AND (“drought” OR “water stress” OR “water scarcity”) AND (“respond” OR “react” OR “influence” OR “impact” OR “effect”)] AND PUBYEAR > 2010 AND PUBYEAR < 2025 AND [LIMIT-TO (DOCTYPE, “ar”)] AND [LIMIT-TO (LANGUAGE, “English”)]
Search query 2	TITLE-ABS-KEY (“plant species” OR “plant community” OR “plant diversity”) AND TITLE-ABS-KEY (“functional trait” OR “plant trait” OR “agronomic trait”) AND TITLE-ABS-KEY (“microbial stimulation” OR “chemoreception” OR “symbiotic”) AND PUBYEAR > 2010 AND PUBYEAR < 2025
Search query 3	TITLE-ABS-KEY [(“endosphere” OR “rhizosphere” OR “bulk soil”)]
Search time frame	Articles published between 2010 and 2025
Boolean operators	“AND”, “OR”
Screening process	PRISMA checklists were followed for the screening process.
Data extraction	Pre-designed data extraction form.

### Assessment and selection of articles

The inclusion criteria were intentionally stringent to ensure the selection of studies with clearly defined microbial responses to drought stress and comparable experimental conditions. While this approach enhances the reliability of comparisons, it may have excluded studies that provide broader contextual insights. The primary literature search terms focused on the publication’s title, yielding an initial collection of 511 publications. These papers underwent further review in pursuit of research that included quantitative and qualitative evaluation of the following: (i) drought-resistant bacteria and fungi; (ii) microbial diversity and abundance under drought stress; and (iii) microbial shift under drought stressed plants. Reviews and book chapters were also included for the study since the experimental research papers that were cited were cross-referenced and assessed for inclusion eligibility. The collected articles were vetted according to a subsequent selection criterion: (i) studies that identified bacteria and fungi to family level; (ii) studies that identified bacteria or fungi when studied in conjunction with plants; (iii) studies that included a positive control (i.e. regular irrigation intervals), and (iv) studies that encompassed forest, field, or greenhouse conditions. Both quantitative and qualitative information regarding the experiment’s location, bacteria, or fungi families/genus/species, plant types/species, experimental conditions and experiment duration were gathered from the carefully curated publications. The literature was reduced to 97 peer-reviewed publications published between 2010 and 2025 after additional screening eliminated duplicate research and those that focused on biotic stressors. Although attempts were made to get translated copies of non-English papers, only the abstracts of papers of relevance were translated. Due to the abstracts’ failure to include bacteria and/or fungi species/genus or identification methods, these studies were subsequently disqualified. With a mechanism-based synthesis across systems shown in Table 2, our thorough review adhered to these steps and standards.

**Table 2:** The criteria for selection of articles for comparative assessment of drought-resilient microbes.

Criteria	Exclusion	Inclusion
Publication	Grey literature including magazines, bulletins and newsletters	Peer-reviewed literature such as experimental original papers, review papers, book chapters, government gazettes and policies
Article	Articles that only had access to abstracts	Full text articles
Abiotic stress	Studies that did not focus on drought-exposed plants/microbes	Plants that focused on drought-exposed plants/microbes
Treatments	Studies that did not include comparative irrigated control	Studies that included comparative irrigated control
Microbes	Studies that did not focus on soilborne drought-resilient microbes	Studies that focused on at least soilborne drought-resilient microbes
Study conditions	Studies that were exclusively conducted under laboratory without inclusion of plants	Studies that were conducted under greenhouse, glasshouse, field and forest with the inclusion of at least one plant species.
Language	Non-English	English
Publication date	Before 2010	After 2010

### Risk of bias assessment

In assessing the changes in bacteria or fungi composition or biomass, bias analysis facilitates insightful comparisons, because it enables us to systematically assess the methodological quality of observational studies. For carrying out risk of bias assessment, the Crowe Critical tool was used to assess different criteria such as design, sampling, and data collection, ultimately categorizing studies into different quality tiers. This ensured that studies were compared using reliable methodologies, thus facilitating accurate interpretations and conclusions regarding the shift in bacteria and fungi and subsequent influence on ecosystem functionality.

### Data extraction and data analysis

**Figure 1:**
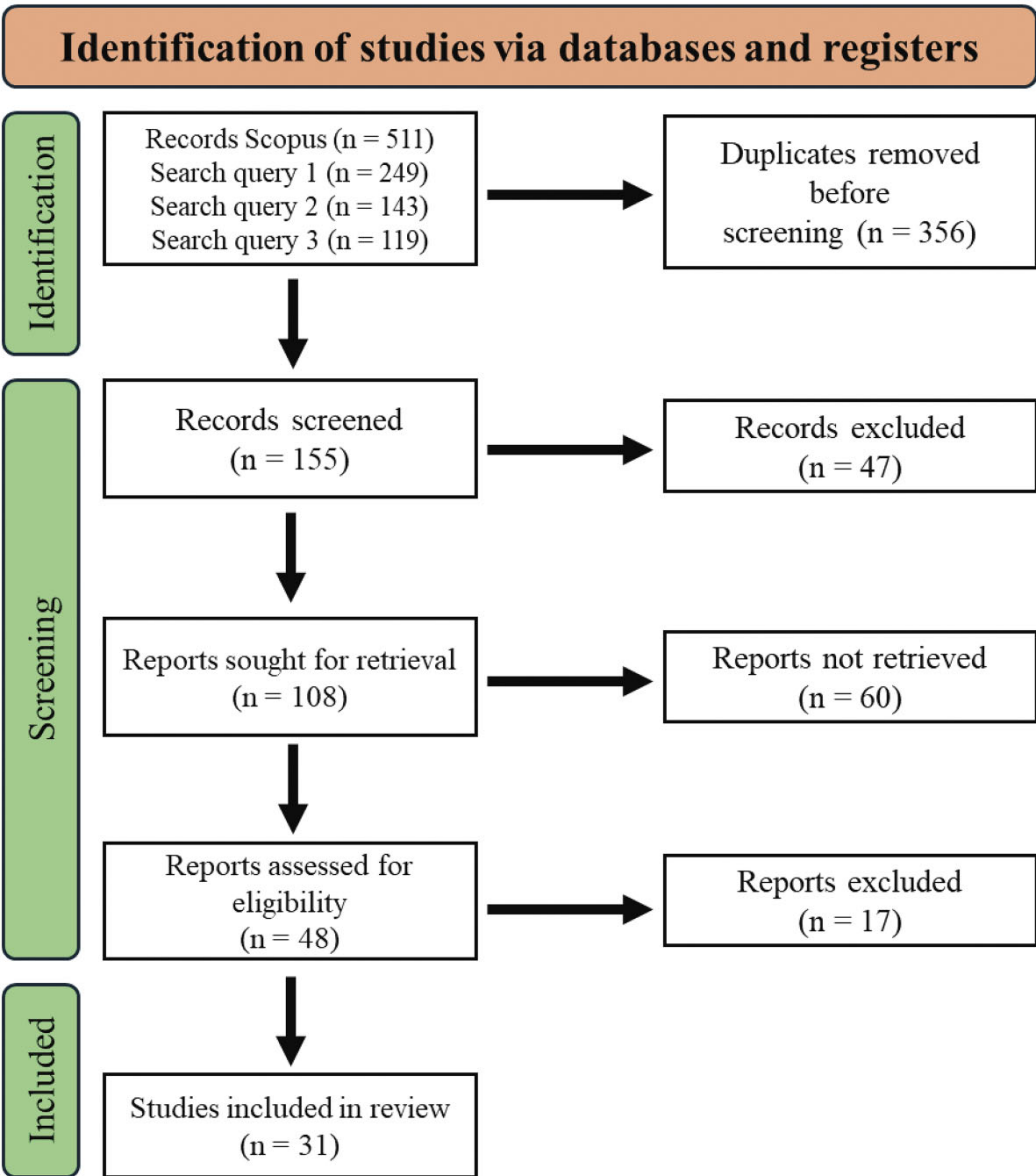
Preferred Reporting Items for Systematic reviews and Meta-Analyses (PRISMA) flow diagram for showing the steps taken for selection of eligible articles for inclusion in the systematic review.

The initial exclusion process involved removing duplicates, resulting in the exclusion of 356 articles. Subsequently, 155 unique articles remained and among these, 47 articles were found ineligible based on their titles and abstracts, as they did not focus on assessing the microbial response to drought. After the screening process, 108 papers qualified for a full-text review. However, 77 articles were excluded during this phase as they did not meet the eligibility criteria established for the review. Ultimately, 31 articles met the selection criteria and were included for data extraction in the study, as illustrated in Fig. 1.

## Results

### Geographical distribution of bacteria response to drought

The reviewed studies cover a wide range of regions with different climates and experimental conditions (Table 3). They included areas in Asia, Europe, Australia, America and Africa, showcasing how drought-resistant microbes are essential in CSA on a scale. Several research studies focused on subtropical areas with a notable emphasis in Asia, where seven studies were conducted (Dai et al. 2019, Ullah et al. 2019, Liu et al. 2021, Xie et al. 2021, Peng et al. 2024, Wu et al. 2024, Halo et al. 2025) on various plants, highlighting Asia’s significance in CSA research within tropical ecosystems. These studies delved into identifying bacteria from plants’ endosphere, rhizosphere and bulk soil The prevalence of China’s influence in the study highlights the importance of advancing towards CSA from microbial perspective in regions where continuous growth leads to ongoing challenges with water scarcity. Studies from temperate regions including United States (Zapata et al. 2021, Yang et al. 2024), Europe (de Souza et al. 2024), and Australia (Maisnam et al. 2023) were also explored in the current review. In these studies, they aimed to assess temporal and spatial microbial shifts under different plant species subjected to drought. In the arid and semi-arid continental climates of Africa, studies including those by (Marasco et al. 2012, Huber et al. 2022, Legeay et al. 2024, Ehinmitan et al. 2025) also identified microbes with potential for mitigating drought stress.

**Table 3:** Spatial and temporal shifts of soilborne bacteria subjected to drought stress on different plant species.

Plant	Objective	Duration	Conditions	Results	Identified bacteria	Location	Source
*Vigna unguiculata* [L.] Walp	To identify the epiphytic rhizobacterial community in cowpea and investigate how their biodiversity responds to drought	9 weeks	Growth room	Compared to the control, epiphytic bacterial communities showed increased diversity on drought-treated cowpea	*Methylophaga, Nocardioides, Paenibacillus, Kangiella koreensis, Halobacteriovorax, Ornithinicoccus halotolerans, Haliea atlantica, Pseudonocardia saturnea, Halocatena pleomorpha, Maricaulis maris, Pelagibacterium halotolerans, Tistlia consotensis, Marinobacter bryozoorum, Thermochromatium tepidum, Streptomyces sediminis*	Oman	(Halo et al. 2025)
*Populus tomentosa* Carrière	To characterise changes in microbes during drought stress	6 months	Forest	Even though overall diversity declined, plant-beneficial fungi were relatively widespread	*Streptomyces rochei, Bacillus arbutinivorans, B. endophyticus, B. megaterium, Aspergillus terreus, Penicillium raperi, Trichoderma ghanense, Gongronella butleri, Rhizopus stolonifer*	China	(Xie et al. 2021)
*Oryza sativa* subsp. *japonica* cv. Jia 67	To explore how drought stress impacts bacterial co-occurring networks	28 days	Field	Drought changed the bacterial community assemblages and raised the Shannon bacteriomes diversity on rice rhizosphere and endosphere	*Sphingomonas, Paracoccus, Mesorhizobium, Streptomyces, Pseudomonas, Exiguobacterium, Anaeromyxobacter*	China	(Wu et al. 2024)
*Gossypium hirsutum* cv. Jin668	To determine microbes that are most suited for cotton’s drought resistance.	−	Greenhouse	Bacterial community significantly increased in the soil with cotton plants compared to fallow	*Sphingomonas, Streptomyces, Gemmatimonas, Sphingopyxis, Acidothermus, Jatrophihabitans*	China	(Ullah et al. 2019)
*Panicum miliaceum* L., *Arachis hypogaea* L.	To study the rhizospheric and bulk soil microbial communities in a cereal/ legume monoculture and intercropping	3 years	Field	Drought was the main factor influencing bacterial composition with no significant differences in monoculture and intercropping	Actinobacteriota, Proteobacteria, Chloroflexi, Acidobacteriota, Firmicutes, Basidiomycota, Ascomycota	China	(Peng et al. 2024)
*Arachis hypogaea* L.	To investigate how peanut developmental stages affect structure the root microbial community after drought	−	Greenhouse	Drastic increase in abundance of Actinobacteria and Acidobacteria in seedling and podding stages, and an increase of Cyanobacteria and Gemmatimonadetes during the flowering stage in drought-treated rhizospheres	*Saccharibacteria, Gaiellales, Sphingomonas, Micrococcaceae, Acidimicrobiales, Microcoleus*	China	(Dai et al. 2019)
*Saccharum officinarum* L.	To investigate the rhizosphere microbial species response to drought stress	3 months	Greenhouse	The change in the bacterial community structure under drought stress had a 25.2% correlation with the drought adaptability of sugarcane	Alphaproteobacteria, Gammaproteobacteria, Sphingobacteriia, Betaproteobacteria, Actinobacteria	China	(Liu et al. 2021)
*Avena barbata* Pott ex Link., *Bromus diandrus* Roth., *Medicago polymorpha* L.	To test how soil microbial diversity, composition and co-occurrence relationships are affected by drought and vegetation restoration	3 years	Field	Compared to soils receiving ambient precipitation, drought dramatically decreased bacterial diversity and composition	Verrucomicrobia, Proteobacteria, Chloroflexi, Cyanobacteria, Planctomycetes, Acidobacteria	United States of America	(Yang et al. 2024)
*Manihot esculenta* Crantz	To isolate bacterial strains from the rhizosphere of cassava subjected to water shortage	31 days	Glasshouse	An increase in the diversity, abundance, and species richness of rhizobacterial community was found in cassava plants subjected to water-deficit stress	*Achromobacter, Acinetobacter, Aeromonas, Buttiauxella, Cronobacter, Klebsiella, Ochrobactrum, Pluralibacter, Pseudomonas, Rhizobium, Serratia, Sphingomonas, Pseudomonas luteola, Ochrobactrum anthropi*	Colombia	(Zapata et al. 2021)
Grasses, herbs, legumes	To examine the changes in bacterial diversity and composition across plant species richness gradient	9 years	Field	Found the taxa that are strongly influenced by drought at each plant richness level and the substantial impact of plant species richness on the makeup of the soil microbial community	Gemmatimonadota, Chloroflexi, Myxococcota, Bacteroidota, Patescibacteria, Actinobacteriota, Planctomycetota, Verrucomicrobiota,	Germany	(de Souza et al. 2024)
Woodland, shrubs	To investigate shifts in the structure of microbial community pre- and post-drought	3 years	Field	Wet periods had high abundances in taxa of Gemmatimonadetes, Acidobacteria, Chytridiomycota. While Actinobacteria, Alphaproteobacteria, Ascomycota, Planctomycetes, Basidiomycota increased during drought	Actinobacteria, Planctomycetes, Ascomycota, Basidiomycota, Alphaproteobacteria	Wales	(Maisnam et al. 2023)
*Malva sylvestris* L.	To determine the structure of microbiome of *M. sylvestris*, in arid regions and evaluate which bacteria are associated with this plant’s drought tolerance	−	Field	The endosphere was dominated by the genus *Rhizobium*	*Rhizobium*, Pseudomonadota, Actinomycetota, Pseudomonadota, Bacillota, Planctomycetota, Chloroflexota,	Morocco	(Legeay et al. 2024)
Woodland, bushveld, fallow	To examine the influence of drought on bacteria and their response to different soil water availability	2 years	Field	High population of Blastocatellia and Vicinamibacteria in loamy sands, while the Acidobacteriia dominated in sandy soils	Acidobacteriia, Blastocatellia, Pyrimonadales, Blastocatellales, Vicinamibacteria	Namibia	(Huber et al. 2022)
*Capsicum annuum* L.	To assess the impact of desert farming on plant-microbe association in pepper cultivated in arid conditions	−	Field	*Bacillus* spp. (68% of the isolates) was mainly recovered from the endosphere, while rhizosphere and the root surrounding soil fractions were dominated by *Klebsiella* spp. (61% and 44% respectively)	*Actinobacteria, Bacilli, Alpha, Pseudomonas, Beta, Gammaproteobacteria*,	Egypt	(Marasco et al. 2012)
*Zea mays* L.	To identify and assess the bacterial strains’ capacity to stimulate plant growth under drought stress	–	Field	Significant improvements in maize vigour index, germination rate, seedling length, height and dry biomass by the combinatorial potential of the identified isolates	*B. cereus, B. velezensis*	Kenya	(Ehinmitan et al. 2025)
*Anthoxanthum odoratum, Dactylis glomerata, Leontodon hispidus, Rumex acetosa*	To evaluate contribution of plant communities in the response of bacterial network and community to drought, as well as the resulting consequences for soil functionality	3 months	Glasshouse	Drought decreased bacterial richness and evenness	Verrucomicrobia, Actinobacteria	England	(de Vries et al. 2018)

### Geographical distribution of fungi response to drought

The reviewed experimental research on fungal diversity during drought also encompassed a broad spectrum of geographical locations with varying weather and experimental setups. They too illustrated the significance of drought-resistant fungi in CSA on a large scale by including regions from Asia, Europe, Australia, America, and Africa (Table 4). Temperate regions were the primary subject of several investigations, with Europe receiving particular attention (de Vries et al. 2018, Schmidt et al. 2018, Lozano et al. 2021, Albracht et al. 2023, Maisnam et al. 2023) on various plants under varying experimental setups. These investigations focused on examining temporal shifts in fungi structure and community under drought on different plants. Studies from United States of America (Hawkes et al. 2011, Buscardo et al. 2021, Carbone et al. 2021, Lagueux et al. 2021, Basyal and Walker 2023, Yang et al. 2024;) were also explored in the current review. These studies aimed to assess temporal and spatial fungal shifts under different plant species subjected to drought, as well as soil functionality. In the tropical and temperate continental climates of Australia, studies including those by (Hopkins et al. 2018, Birnbaum et al. 2019) and Asia (Wang et al. 2024a,b, Andreo-Jimenez et al. 2019, Maitra et al. 2019) identified fungi with potential for mitigating drought stress under different experimental settings across greenhouse, field and forests. These studies established that fungi are sensitive to drought stress, with more abundance of arbuscular mycorrhizal fungi (AMF), ectomycorrhizal fungi (ECM), and saprophytes.

**Table 4:** Spatial and temporal shifts of soilborne fungi subjected to drought stress on different plant species.

Plant	Objective	Duration	Conditions	Results	Identified fungi	Location	Source
Woodland, shrubs	To investigate shifts in the structure of microbial community pre- and post-drought	3 years	Field	Fungi were shown to be more sensitive to the prolonged drought and to rainfall treatment than bacteria with Basidiomycota mostly dominant in the reduced rainfall treatment	Ascomycota, Basidiomycota, Chytridiomycota, Mortierellomycota	Wales	(Maisnam et al. 2023)
*Eucalyptus marginata, Corymbia calophylla*	To measure the rhizosphere fungal communities in a drought impact gradient	3 years	Forest	Fungal community composition and abundance of functional groups were altered in the soil underneath the drought-affected tree, with less ECM mycorrhizal fungi and greater levels of AMF and saprotrophs	Arbuscular mycorrhiza, ectomycorrhizal fungi, ericoid, saprophytes, endophytes	Australia	(Hopkins et al. 2018)
Proteaceae, Fabaceae, Myrtaceae,Ericaceae	To assess how warming and drying conditions shape soil fungi	15 months	Forest	When compared to control plots, soil fungal diversity increased during warming, suggesting that warming had a beneficial impact on fungal diversity	Endophytes, ericoid mycorrhizas, yeasts, ectomycorrhizas	Australia	(Birnbaum et al. 2019)
Grasses, herbs, legumes	To determine the long-term impacts of drought on interactions between fungi and plants	9 years	Field	Shifts in AMF and overall fungal community structures brought on by recurring drought	*Diversispora torrecillas, Penicillium jenensii, Paraglomus* spp.,	Germany	(Albracht et al. 2023)
Grasses	To assess how different moisture conditions affect overall fungal diversity, community composition and functionality	15 weeks	Grassland	Changes in community structure, but richness of communities was not changed and decrease in the relative abundance of pathogens	Sordariomycetes, Agaricomycetes, Eurotiomycetes	Germany	(Schmidt et al. 2018)
*Avena barbata, Bromus diandrus, Medicago polymorpha*	To test how soil microbial diversity, composition and co-occurrence relationships are affected by drought and vegetation restoration	3 years	Field	Fungal diversity and composition were not significantly impacted by either vegetation restoration or drought	Ascomycota	United States of America	(Yang et al. 2024)
*Anthoxanthum odoratum, Dactylis glomerata, Leontodon hispidus, Rumex acetosa*	To evaluate contribution of plant communities in the response of fungal networks and community to drought, as well as the effects of drought on soil fungi and the resulting consequences for soil functioning	3 months	Glasshouse	Fungal richness and evenness increased during the drought period	Ascomycota, Glomeromycota	England	(de Vries et al. 2018)
*Andropogon gerardii, Bouteloua curtipendula, B.dactyloides, B. eriopoda, B. gracilis, Schizachyrium scoparium*	To assess the effects of drought on grassland root-associated fungi and to investigate the differences between these grasslands in the fungi linked to the major grasses	5 months	Greenhouse	Fungal diversity and abundance in roots were mostly unaltered by drought, but the fungal community makeup was significantly rearranged	*Rhizophagus, Podospora, Lophiostoma, Preussia, Cladophialophora, Marasmius, Lachnum, Marchandiomyces, Mortierella, Chaetomium, Monosporascus*	Central United States	(Lagueux et al. 2021)
*Panicum virgatum* L.	To investigate how AMF species affect switchgrass physiology and growth during periods of severe drought	3 months	Greenhouse	The aboveground biomass of mycorrhizal plants grew more than three times under drought compared to the uninoculated control plants, despite the fact that drought drastically reduced mycorrhizal colonization by about 37%	*Claroideoglomus etunicatum*	United States of America	(Basyal and Walker 2023)
*Schima superba, Castanopsis fargesii, Castanopsis carlesii, Lithocarpus glaber, Hicriopteris glauca*	To examine the response of AM fungi to drought in different seasons	4 years	Forest	Drought significantly affected AM fungal extra-radical hyphal density, spore density and root colonization rate	Glomerales, Diversisporales, Archaeosporales, Paraglomerales	China	(Maitra et al. 2019)
*Medicago sativa* “Qingshui”, “Longdong”, *M. varia* “Gongnong”	Looking at which microbes are best for alfalfa’s drought resistance and how drought stress alters the endogenous hormone content of the root system	4 months	Greenhouse	Different alfalfa species promoted different relative abundance of fungi	Ascomycota, Dothideomycetes, Capnodiales, Cladosporiaceae, *Cladosporium, Cladosporium sphaerospermum*	China	(Wang et al. 2024a)
*Castanopsis fargesii, Castanopsis sclerophylla, Lithocarpus glaber, Schima superba*	To investigate how long-term drought affects the microbial population at various soil depths	7 years	Forest	While Basidiomycota increased with increasing soil depth, Ascomycota relative abundance declined. On fungal species including Chytridiomycota and Rozellomycota, there were notable interactions between soil depth and drought. Even though Kickxellomycota are known to be less abundant in soil, their relative abundance was greatly enhanced by drought	Basidiomycota, Kickxellomycota	China	(Wang et al. 2024b)
Grassland	To examine how soil fungi respond to rain seasonal changes in a grassland ecosystem	5 years	Field	Rainfall levels had a direct impact on fungi, with abundant, varied, and stable communities growing under drought and less abundant, varied, and unstable communities during wetter times	Ascomycota, Basidiomycota, Glomeromycota, Chytridiomycota	United States of America	(Hawkes et al. 2011)
Grasses, forbs, legumes	To evaluate how plant species (i.e. grasses, forbs, legumes) react to drought in terms of fungal communities and the functional groups linked to their roots	3 months	Field	Root characteristic adaptations to water shortage were linked to the fungal response to drought, which was strongly dependent on the fungal group	*Gibberella tricintata, Urocystis agropyri, Powellomyces hirtus, Mortierella minutissima,Mortierella sarnyensis, Trichocladium opacum, Preussia flana-ganii*	Germany	(Lozano et al. 2021)
*Vitis vinifera* L.	To explore the impact of drought on root-associated fungal communities	6 and 12 months	Greenhouse	All three compartments' total fungal composition was considerably altered by drought, with the root endosphere compartment varying from the well-watered control the most.	Nectriaceae, Mortierellaceae, Ceratobasidiaceae, Mortierellaceae, Ceratobasidiaceae, Mortierellaceae,	Uruguay	(Carbone et al. 2021)
*Oryza sativa* ssp. indica	To examine how drought affects fungal microbiomes associated with the roots of several rice cultivars and if these alterations may help rice withstand drought	4 months	Field	Drought enhanced fungal diversification and changed the makeup of the fungal community associated with roots	Glomeromycota, Ascomycota, Zygomycota, Basidiomycota, Chytridiomycota,	Philippines	(Andreo-Jimenez et al. 2019)
Evergreen rainforest	To investigate the effects of natural seasonality in an Amazonian rain forest with 14 years of partial throughfall exclusion, with an emphasis on the functional diversity of soil fungi	14 years	Forest	Drought causes significant changes in fungal diversity and functional group composition, with noticeable rise in dark-septate fungal abundance and a decline in fungal pathogens	Ascomycota, Rozellomycota, Basidiomycota, Mucoromycota, Chytridiomycota, Entomophthoromycota, Glomeromycota, Mortierellomycota	Brazil	(Buscardo et al. 2021)

AMF: arbuscular mycorrhizal fungi, EMC: ectomycorrhizal fungi

## Discussion

### Soil microbial diversity and abundance under drought

Based on the reviewed studies, a conceptual framework emerges in which plant–microbe interactions under drought stress are mediated by interconnected mechanisms. These include (i) plant-driven root exudation that selectively recruits beneficial microbes, (ii) microbial enhancement of plant stress tolerance through osmolyte production and phytohormone regulation, and (iii) structural modifications such as enhanced root growth that improve water acquisition. While the relative importance of these mechanisms varies across systems, they collectively represent a generalizable pathway through which plants and microbes interact under water-limited conditions.

Different plant species have shown varying effects of drought on the variety of rhizospheric bacteria, and this diversity can occasionally be influenced by the host plant species, soil conditions, and the intensity of the applied drought (Moore et al. 2023). Drought, for example, decreased microbial diversity in other plant species, including *Populus* trees (Xie et al. 2021), but increased microbial diversity in the rhizosphere of cowpea (Halo et al. 2025). In rice, the co-occurrence network in the rhizosphere became more complicated due to drought circumstances. Bacterial diversity, as shown by the Shannon index, rose while species richness stayed the same (Wu et al. 2024). Studies such as those by Wu et al. (2024) indicated differences in microbial diversity differences across endosphere, rhizosphere and bulk soil. Drought stress boosted bacterial alpha diversity in cotton rhizospheres, and some bacterial phyla, including Chloroflexi and Gemmatimonadetes, were enriched (Ullah et al. 2019). Similarly to bacteria dynamics under drought, fungi composition and diversity are also influenced by drought periods and plant species. In a study by Hopkins et al. (2018), the rhizospheric soil of *Eucalyptus marginata* and *Corymbia calophylla* trees damaged by dryness had a different fungus community composition and functional group abundance, with more AMF and saprotrophs and less ECM. In the drought-affected areas, fungal communities under live and dead plants differed as well; under dead trees, the drought-affected plots had more AMF and saprophytes, but a decline in pathogenic fungal species (Schmidt et al. 2018, Buscardo et al. 2021). Under greenhouse conditions, while fungal diversity and abundance in roots were mostly unaltered by drought, the fungal community composition was significantly rearranged on different grass species subjected to drought stress (Lagueux et al. 2021).

The plant holobiont is made up of the rhizosphere and endosphere microbes, which are essential for changing the composition of soil nutrients and for facilitating plants tolerating a wide range of biotic and abiotic stressors, including drought. The distinction between rhizosphere and endosphere microbial communities is well established, with each niche hosting structurally and functionally distinct microbiomes (Trivedi et al. 2020). This was further supported by Wu et al. (2024), who showed that the dominant bacterial taxa and community structure in the rhizosphere and endosphere of *O. sativa* were significantly different from those in the bulk soil under drought. In this study there were notable enrichment of Gemmatimonadetes, Patescibacteria, and Actinobacteriota, while populations of Firmicutes, Bacteroidetes, and Desulfobacterota, declined in the rhizosphere and endosphere (Wu et al. 2024). Similarly, Ullah et al. (2019) established differences in bacteria diversity of *G. hirsutum* rhizosphere and bulk soil, where drought treatment reduced the abundance of Streptomyces but increased the abundance of Sphingomonas. Dynamic changes were seen in the rhizosphere communities during the drought treatment. The relative abundance of Actinobacteria and Alphaproteobacteria in the rhizosphere bacterial community increased, while the relative abundance of Betaproteobacteria and Sphingobacteria decreased (Liu et al. 2021).

Plant growth and overall health have been shown to be impacted by drought-induced alterations in microbial populations. When assessing the impact of desert farming on plant-microbe association in pepper cultivated in arid conditions Marasco et al. (2012) discovered that *Bacillus* species (68% of the isolates) was mainly recovered from the endosphere, while rhizosphere and the root surrounding soil fractions were dominated by *Klebsiella* species (61% and 44%, respectively). When treated with drought after inoculation, the plants’ growth was higher than that of the groups that received regular watering (Xie et al. 2021). In yet another study, Peng et al. (2024) established that the primary factor influencing the makeup of microbial communities was drought, which led to a decrease in the abundance of Proteobacteria, Acidobacteriota, and Ascomycota, while it resulted in an increase in Actinobacteriota, Firmicutes, and Basidiomycota. Halo et al. (2025) identified species previously identified in saline environments such as *Adhaeretor mobilis, Halobacteriovorax* spp., *Haliea atlantica, Halocatena pleomorpha, Kangiella koreensis, Marinobacter bryozoorum, Maricaulis maris, Ornithinicoccus halotolerans, Pelagibacterium halotolerans*, and *Tistlia consotensis*. The species list also includes bacterial species previously identified in desert habitats, such as *Nocardioides* spp., *Pseudonocardia saturnea*, and *Streptomyces sediminis*, as well as in the hot spring, such as *Thermochromatium tepidum*.

In a microbial co-occurrence study de Vries et al. (2018) established that drought predominately encourages altering characteristics in networks of soil bacteria more than that of fungal species, and that alterations in bacterial communities have a stronger correlation with soil functioning following recovery than do alterations in fungal communities. In a study by Hopkins et al. (2018), along the drought effect gradient, the composition of fungal communities varied considerably, with drought-affected stands having lower levels of diversity. While there was no difference in taxonomic richness and diversity, there was considerable indication of community divergence between dead and living trees. In comparison to surviving trees from the unaffected plots, die-off-impacted plots contained more saprotrophs and AMF and less ECM when analyzed by functional group. Additionally, dead and living tree rhizosphere samples in die-off plots had lower ECM, higher AMF, and saprotrophs and pathogens. Fungal diversity and colonization were mostly resistant to drought, although species relative abundances were rearranged by dryness, changing the makeup of the root fungal community. Of the fungus linked with roots, the grass variety that showed the most decreases under drought had the least amount of reordering. In response to changed precipitation, plants may benefit from a stabilizing mechanism that is provided by the reorganization of the fungal population associated with the roots (Lagueux et al. 2021). This implies that rather than plant-associated fungi directly responding to climatic factors, the biggest changes in plant-associated fungi will probably be brought about by changes in plant relative abundance or the extinction of plant species.

### Plant species facilitate soil microbial assemblages under drought

As much as microbes facilitate plant growth, to an equitable degree, plant species also stimulate the establishment of certain microbial communities. Plant-microbial community is facilitated by root exudates, which are also essential for ecosystem response to environmental change (Williams and de Vries 2020). Under moderate drought, plants often boot carbon allocation to roots and release osmoprotectants like proline, trehalose, and glycine betaine to stabilize cellular functions. As drought intensifies, plant metabolism shifts from primary to secondary metabolites such as flavonoids, caffeic acid, and lignin precursors to protect tissues, counter oxidative stress, and signal beneficial microbes. Specific peptides and antioxidants including acacetin and luteolin accumulate during severe drought to maintain turgor pressure and enhanced drought tolerance. Regardless of recovery, drought exposure boosted host growth in C and N metabolic pathways (amino acids, fatty acids, and phenolic glycosides), according to plant metabolomic analysis. Several metabolites showed a favorable correlation with microbial alpha-diversity linked with roots (Veach et al. 2020). Compared to irrigated controls, the composition of the soil bacterial community changed when *P. trichocarpa* and drought were present, but only when plants were present did the composition of the soil fungal community change. On the other hand, root bacterial populations altered less with dryness than root fungal communities (Veach et al. 2020). Likely for increased investment in root growth due to reduction or absence of photosynthetic capacity (Gargallo‐Garriga et al. 2015). The buildup of organic or amino acid metabolites in the roots, which was higher in plants experiencing drought, may draw microbial endophytes (Andreo-Jimenez et al. 2019) and provide characteristics that promote plant development (Rolli et al. 2015). Though they did not identify plant roots exudates, Lozano et al. (2021) concluded that there were plant species-specific differences in fungal beta diversity between the control and drought conditions. While mutualistic fungi exhibited the reverse tendency, saprotrophic fungi for some species increased in relative abundance and richness with drought. And while it was minimally impacted by drought, the community structure of pathogenic fungus was specific to plant species.

The presence or absence of plants play significant roles in the abundance and diversity of soil microbial shift under drought conditions. According to taxonomic study by Ullah et al. (2019), the phyla Proteobacteria (31.7%), Actinobacteria (29.6%), Gemmatimonadetes (9.8%), Chloroflexi (9%), Cyanobacteria (5.6%), and Acidobacteria made up the majority of the bacterial community structure in the cotton rhizosphere subjected to drought. Though this study did not identify roots exudate, the diversity and rise in bacteria population on cotton than in fallow means that plants stimulate bacterial composition. A similar trend was observed in other studies including (Dai et al. 2019, Zapata et al. 2021, Halo et al. 2025). Although bacteria from the surrounding soil environment are drawn to root exudates, notable variations in the makeup of microbial communities across various plant species have been discovered. Findings by Ullah et al. (2019) further demonstrated that the alpha diversity of the rhizosphere bacterial community is affected differently by cotton roots in drought-affected and normal conditions. Likewise, Wang et al. (2024a) reported that *Medicago* species subjected to drought had different fungal abundance. In their study, Basidiomycota, Tremellomycetes, Filobasidiales, Filobasidiaceae, and *Filobasidium oeirense* increased in *M. sativa* “Qingshui” and decreased in *M. sativa* “Longdong” and *M. varia* “Gongnong”. The relative abundance of Sordariomycetes, Hypocreales, and Nectriaceae decreased in *M. sativa* “Qingshui” and increased in *M. varia* “Gongnong”. On the other hand, Ascomycota, Dothideomycetes, Capnodiales, Cladosporiaceae, *Cladosporium*, and *Cladosporium sphaerospermum* increased initially before declining and were at their lowest in all *Medicago* species under moderate stress.

### Microbial assemblages influence plant adaptability to drought stress

Though these reviewed studies suggest a shift toward drought-tolerant micro-organisms, there is a general trend of a decline in total bacteria diversity in other studies (Xie et al. 2021). Nonetheless, microbial diversity does not signify reduced microbial functionality. By reducing diversity and altering the abundance of certain microbial species, drought dramatically changed microbial ecosystems in support of those that facilitate drought resilient (Liu et al. 2021). Beneficial microbes can encourage the creation of osmolytes, certain exudates, or peroxidase, which eliminates reactive oxygen species that are common during water stress (Allard-Massicotte et al. 2016). Under drought and salinity stress, plant phytohormone levels, particularly auxins such as indole-3-acetic acid (IAA), may decline; however, this can be compensated by IAA-producing rhizobacteria such as salt-tolerant *Azospirillum brasilense* NH, which enhance plant growth and stress tolerance (Nabti et al. 2010). Halo et al. (2025) found that the most prevalent genus on cowpea under drought stress was Nocardioides. Because they can break down a variety of chemical substances, including those that are persistent contaminants in the environment, Nocardioides species are significant to the ecosystem health and functionality. Drought-tolerant microbes are renowned for aiding in the natural biodegradation and bioremediation processes by decomposing hydrocarbons and aromatic chemicals. Their ecological relevance is further highlighted by the fact that they may flourish in a variety of settings, including those with low nutrient levels and even some polluted areas. In studies similar the one conducted by Marasco et al. (2012), well-irrigated plants that had been inoculated with the rhizobacterial suspensions were abruptly subjected to a twelve-day water stress phase, demonstrating the potential of these species to support CSA. In the same study, control plants suffered significant damage following eight days of water stress, but plants inoculated with rhizobacteria showed increased shoot turgor.

Through a mutualistic interaction, microbial communities assist plants in fending off and recovering from abiotic challenges, increasing agricultural output and resilience (Liu et al. 2021). For example, *Azospirillum* species in the rhizosphere improved plant water-use efficiency and nutrient uptake during drought conditions by producing osmolytes (e.g. proline and trehalose) and by stimulating root growth, thereby enhancing access to water in deeper soil layers (Prisa and Fresco 2023). Chloroflexi and Gemmatimonadetes are aerobic/anaerobic thermophilic bacteria that thrive in drought environments, as has been extensively described (Ward et al. 2018). Accordingly, these groups of bacterial phyla may improve their resistance to drought by using chloroflexi and gemmatimonadetes to support a variety of plant physiological processes during drought stress. To ascertain the role of plants in facilitating soil microbiota resilience to water stress, *Populus trichocarpa* colony and bulk soils without plants were subjected to prolonged drought (∼0.03% gravimetric water content), rewetting, and a 12-day recovery phase. Drought stimulation augmented host accumulation in C and N metabolic pathways (amino acids, fatty acids, phenolic glycosides), according to plant metabolomic analysis. In contrast to irrigated controls, the makeup of the soil bacterial community changed in response to *P. trichocarpa* and drought, while the composition of the soil fungal community changed only in response to plant presence (Veach et al. 2020). Sequences linked to metabolism, signaling transduction, defense mechanisms, and fundamental essential activities were shown to be enriched in the drought-treated rhizosphere, according to metagenomic profiling (Dai et al. 2019). This finding may have consequences for plant survival and drought tolerance.

## Research gaps and prospects

The findings of this review should be interpreted in light of certain limitations. The included studies represent a wide range of geographic regions, plant species, and experimental conditions, which introduce heterogeneity and may limit direct comparability. Additionally, the relatively small number of studies (n = 31) means that some observations are based on a limited number of cases. As such, the conclusions drawn primarily reflect general trends rather than universally applicable outcomes. The reviewed studies varied considerably in taxonomic resolution, with some identifying microbes at species level, while others reported only genus- or family-level classifications, limiting the ability to accurately infer functional roles. The identification to phyla (Peng et al. 2024, Yang et al. 2024) and genus (Dai et al. 2019) level could prove ecotoxic as species from the same genus could have positive and negative impact on the same plants. There are both positive and negative impacts that *Aspergillus* species may have on plants. While some species are plant pathogens, which cause diseases and financial losses, others are helpful, which through a variety of processes promote plant growth and health (Ramatsitsi et al. 2023). Plant root exudates selectively stimulate specific bacteria and fungi to colonize roots, thereby shaping rhizosphere microbial communities (Hartmann et al. 2009). Moreover, in such studies, it could be argued that less accurate conclusions could be drawn. For instance, the study by Yang et al. (2024) concluded that both drought and vegetation had no impact of fungi composition. However, this study only identified fungi to phylum level, Ascomycota. Within this phyla hundreds of fungal species have been identified, some pathogenic and some beneficial. As such, though overall composition may not have been significantly impacted, it is possible that there were significant effects of fungi diversity. Species level identification further allows for inoculation of isolates that have been identified to not only tolerate drought but also improve plant growth under drought stress. There have been fewer studies such as thobe by Cao et al. (2022) and Wang et al. (2024b), that have inoculated known drought-tolerant species to increase crop growth. Advancement of research in this area would immensely assist the promotion and application of soil microbes in CSA in regions where water scarcity affects crop productivity. Conversely, cultivating plants, through intercropping, that facilitate sporulation of drought-tolerant microbes could be practiced improving growth of plants that are affected by water scarcity. This is especially true in cases where the inoculated drought-resistant microbes cannot be sustained by the cultivated plant, as it was clear in the current review that different plants promote different groups of microbes.

The reviewed papers provide an indication that plants can survive drought periods by influencing micro-organisms that live in the rhizosphere. Studies including those by Pivato et al. (2021), noted differences in microbial diversity between rhizospheric and bulk soil, indicating that these plants possibly formed a symbiotic relationship with drought-resilient microbes. However, limited studies have explicitly evaluated plant root exudation under drought stress. It is well established that, under stress conditions, plants release specific metabolites that selectively stimulate microbial communities associated with stress mitigation (Hartmann et al. 2009). For instance, drought elevated the exudation of organic acids in maize, especially malic acid (together with fumaric, malonic, succinic, and oxalic acids (Henry et al. 2007), which is a powerful chemoattractant for *B. subtilis* (Allard-Massicotte et al. 2016). Through the activation of osmolyte secretion, the beneficial bacterial species *B. subtilis* promotes drought tolerance in *Phleum pratense* L. (Gagné-Bourque et al. 2016). *Pseudomonas putida* and *B. amyloliquefaciens* are rhizosphere bacteria in chickpea that mitigate the effects of drought (Kumar et al. 2017). Although the precise chemical signals mediating these interactions are not fully understood, root exudates are widely recognized as key drivers of plant–microbe interactions, functioning as signaling molecules, chemoattractants, and nutrient sources for beneficial microbes (Hartmann et al. 2009). Plant responses to drought may therefore be partly mediated by species-specific changes in root exudation patterns, which influence the structure and function of associated microbial communities. These plant–microbe interactions can, in turn, affect plant recovery and broader ecosystem functioning. Even though there are still a lot of unanswered questions, the data makes it abundantly evident that changes in root exudation patterns brought on by drought might affect rhizosphere functions and perhaps affect how an ecosystem responds to drought. Through metabolomic profiling of root exudates in conjunction with focused evaluations of bacterial and fungal communities, advancing towards CSA that addresses productivity arid and semi-arid regions could be achieved.

Limited data on how soil management practices (cover crops, reduced tillage, organic amendments) influence microbial resilience to drought. The incorporation of proper production practices to foster microbial communities that can maintain soil functionality under water stress. By increasing the diversity, quantity, and activity of soil micro-organisms, soil management techniques can greatly increase their resistance to drought. Beneficial micro-organisms’ capacity to tolerate and recover from drought stress can be increased by employing techniques including conservation agriculture, the application of organic amendments, and strategic nutrient management. In other studies, such as Lozano et al. (2021), wherein there was considerable abundance of pathogenic fungal species, conservation agriculture could promote beneficial micro-organisms as this system has been reported to promote beneficial soil microbes. For instance, by minimizing soil disturbance through reduced or no-tillage techniques, beneficial microbial populations can thrive in a more stable environment that maintains soil structure, lowers erosion, and conserves moisture (Xia et al. 2019). The same is true for using cover crops and incorporating crop residue. Crop residues retained on the soil’s surface offer an organic matter supply that supports microbial activity and enhances soil structure, improving microbial resilience and water retention. Cover crops, particularly those with deep roots, can improve soil structure, boost microbial diversity, and promote water penetration, all of which improve drought tolerance (Taskin et al. 2021). *Pseudomonas, Burkholderiales*, and *Rhizobiales* are some examples of beneficial micro-organisms that are known to increase plant growth and improve drought tolerance and are fostered by conservation agricultural approaches (Kost et al. 2024).

## Concluding remarks

Since water availability is clearly a major limiting factor in plant output, the health, and functionality of agroecosystems may be particularly vulnerable to climate change. Drought is an abiotic stress that modifies the soil environment and impact soil organisms, which is a substantial, concerning, and dangerous circumstance. The activity and functional composition of soil bacteria and fungi that oversee essential ecosystem services and functions are changed. Plant anatomy, physiology, biochemistry, and morphology are all directly impacted by drought. The synergy that exists between plants and microbes can be mediated to improve drought tolerance. It is imperative to note that rhizosphere microbial communities have potential to lessen the detrimental impacts of drought on plants by modifying their structural and functional makeup in response to water shortage. According to the reviewed literature, drought stress dramatically changes the diversity and quantity of epiphytic and rhizosphere bacterial and fungal communities, which affects the soil ecological balance and plant performance. The findings demonstrate how microbial resistance to drought promote plant resilience by mitigating water shortage stress, offering valuable insights for promoting CSA and creating microbial-based techniques to increase crop yields in areas vulnerable to drought. In the face of climate change, in particular, soil microbial populations play a crucial role in promoting resilience and resistance in ecosystems, thereby restoring soil functionality and maintenance of plant health. Microbiomes support plant health, which helps restore ecological processes such as carbon sequestration and nutrient cycling and lessens the impact of climate change. Improved plant resilience to drought stress is another way that microbiomes lessen the need for advanced irrigation systems. These drought-adapted species’ phylogenetic grouping raises the possibility that their capacity to endure drought environments is linked to evolutionary ties. These results demonstrate how the rhizospheric microbiome may contribute to plant drought tolerance, which may have implications for farming methods including the production of biofertilizer for areas that are prone to drought.

Under drought conditions, microbial diversity and biomass often declines, fragmenting soil microbial networks and reducing ecosystem functionality. Nonetheless, spore-forming and exopolysaccharide-producing bacteria and fungi, such as *Bacillus* and *Streptomyces*, tend to increase in relative abundance due to their enhanced tolerance to osmotic stress. Drought favors drought-resilient bacteria and fungi phyla such as Actinobacteria, Firmicutes, and Chloroflexi, which possess structural and metabolic adaptations for low water environments. Despite reductions in microbial richness, these stress-adapted taxa maintain essential functions such as nutrient cycling, growth promotion, and stress mitigation. Endophytic communities are often more affected than rhizospheric ones, as their survival is closely tied to plant internal physiology and water status. Microbial shifts under drought correlate with changes in plant metabolome profiles, reflecting a coordinated response between plants and microbiomes. Plants dynamically alter the quantity and composition of root exudates in response to drought, with patterns influenced by species identity, drought severity and time. Moreover, plant resistance to drought may be influenced by the prevalence of drought-tolerant ectomycorrhizal species, such as *Cenococcum geophilum*, or the transition of mycorrhizal groups from ectomycorrhiza to arbuscular mycorrhizae fungi. Supporting local and global initiatives for promoting sustainable agriculture to provide food and nutrition security while including essential adaptation and mitigation is the overarching goal of CSA. To achieve this goal, three primary objectives are established: (i) increasing agricultural productivity in a sustainable manner; (ii) adapting and strengthening resilience to climate change at the farm and national levels; and (iii) creating opportunities to lower greenhouse gas emissions from agriculture in comparison to historical trends. It is not implied that every practice used in every site should provide these “threefold wins”, even while CSA strives to achieve all three goals. While these findings highlight important trends, further research across diverse systems is required to strengthen the generalizability of these conclusions.
